# Versorgung gefäßchirurgischer Patienten während COVID-19: eine deutschlandweite Umfrage

**DOI:** 10.1007/s00772-022-00871-8

**Published:** 2022-03-04

**Authors:** Georg Jung, Maria-Elisabeth Leinweber, Farzin Adili, Thomas Schmitz-Rixen

**Affiliations:** 1 Universitätsklinikum Frankfurt am Main, Klinik für Herz- und Gefäßchirurgie, Goethe-Universität Frankfurt am Main, Theodor-Stern-Kai 7, 60596 Frankfurt am Main, Deutschland; 2 Klinik für Gefäßmedizin – Gefäß- und Endovascularchirurgie, Klinikum Darmstadt, Darmstadt, Deutschland; 3 Deutsches Institut für Gefäßmedizinische Gesundheitsforschung (DIGG) gGmbH, Berlin, Deutschland

**Keywords:** Personalbelastung, Versorgungsforschung, Versorgungsqualität, OP-Kapazität, Pandemie, Staff load, Health services research, Quality of care, OP capacity, Pandemic

## Abstract

**Hintergrund:**

Durch COVID-19 kam es weltweit, insbesondere in den ersten Wochen der Pandemie, zu einer Verschiebung und Absage elektiver Operationen in allen chirurgischen Fachdisziplinen. Eine Beschreibung der spezifischen Situation in gefäßchirurgischen Kliniken in Deutschland während dieser Periode ist bislang nicht erfolgt.

**Ziel der Arbeit:**

Zweck der Befragung war die Erfassung der gefäßchirurgischen Leistungserbringung in der Zeit von März 2020 bis Dezember 2020, sowie von logistischen und infrastrukturellen Veränderungen, die sich durch die pandemische Lage ergeben hatten. Hierbei lag der Fokus der Umfrage auf der möglichst realitätsnahen Abbildung der Versorgungssituation anhand der Einschätzung der leitenden Gefäßchirurg*innen.

**Material und Methoden:**

In Zusammenarbeit mit der Deutschen Gesellschaft für Gefäßchirurgie und Gefäßmedizin (DGG) wurde das leitende ärztliche Personal von gefäßchirurgischen Einrichtungen in Deutschland aufgefordert, an der Umfrage teilzunehmen. Die Beantwortung der Fragen erfolgte anonym.

**Ergebnisse:**

Durch COVID-19 und korrespondierende Maßnahmen kam und kommt es zu relevanten Absagen und Verschiebungen von Operationen, Verlust an Kapazitäten und einer gesteigerten Personalbelastung. Es traten im Beobachtungszeitraum verspätete Versorgungen gefäßchirurgischer Krankheitsbilder und ein gehäuftes Auftreten schwererer klinischer Stadien verglichen mit dem entsprechenden Vorjahreszeitraum auf. Betroffen sind alle Versorgungsstufen, größtenteils dauern diese Veränderungen an.

**Diskussion:**

Um der strukturellen Schwächung und den Einschränkungen in der Patientenversorgung zu begegnen, sind klinische Abläufe, Patientenaufklärung und Priorisierung zu optimieren. Neue Konzepte wie z. B. Telemedizin und engmaschigere klinische Kontrolle sind ggf. sinnvoll. Eine erforderliche Infrastruktur für Notfallmanagement (COVID) darf im Alltag nicht die Versorgungsqualität der gefäßchirurgischen Patient*innen negativ beeinflussen.

**Zusatzmaterial online:**

Die Online-Version dieses Beitrags (10.1007/s00772-022-00871-8) enthält den Fragebogen zur Umfrage „Versorgungsrealität gefäßchirurgischer Patienten während der COVID-19-Pandemie“.

## Abstract (en)

**Background:**

COVID-19 resulted in postponement and cancellation of elective operations in all surgical disciplines worldwide, especially in the first weeks of the pandemic. The specific situation in vascular surgery clinics in Germany during this period has not yet been described.

**Objective:**

The purpose of the survey was to record the provision of vascular surgery services in the period from March 2020 to December 2020 as well as logistical and infrastructural changes that resulted from the pandemic situation. The focus of the survey was on mapping the supply situation as realistically as possible based on the assessment of the lead vascular surgeons.

**Material and methods:**

In cooperation with the German Society for Vascular Surgery and Vascular Medicine (DGG), the senior medical staff of all vascular centers in Germany were asked to take part in the survey. The questions were answered anonymously.

**Results:**

COVID-19 and corresponding measures resulted in relevant cancellations and postponements of operations, a loss of capacities and an increased workload. During the observational period there was a delay in the treatment of vascular surgical diseases and an increased occurrence of severe clinical stages compared to the corresponding period of the previous year. All levels of care were and continue to be affected.

**Discussion:**

In order to counteract the structural weakening and the restrictions in patient care, clinical processes, patient education and prioritization must be optimized. New concepts such as telemedicine and closer clinical control may make sense. A necessary infrastructure for emergency management (COVID) must not negatively affect the quality of care for (vascular) surgical patients in everyday life.

## Hintergrund und Fragestellung

Aufgrund der rapiden Zunahme der Fallzahlen außerhalb Chinas erklärte der WHO-Generaldirektor am 11. März 2020 den Covid-19-Ausbruch offiziell zu einer Pandemie, Mitte März 2020 war Europa zum Epizentrum der Pandemie geworden und meldete über 40 % der weltweit bestätigten Fälle.

Aus der Ex-post-Perspektive wurden im März 2020 beinahe ausschließlich Maßnahmen eingeleitet, die darauf fokussierten, die Verbreitung der Erkrankung einzudämmen. Entsprechend konzentrierte sich die Berichterstattung auf epidemiologische und statistische Kennzahlen. Neben Kontaktbeschränkungen und Teil-Lockdown im öffentlichen und privaten Rahmen war ein Schwerpunkt der frühen Maßnahmen in den Krankenhäusern die Bereitstellung von Intensivkapazitäten und ausreichender Schutzausrüstung für das medizinische Personal [1].

Diese Entwicklung hatte zur Folge, dass die föderale bzw. kommunale Exekutive eine Absage bzw. Verschiebung elektiver Operationen einforderte, ohne dabei weitere Empfehlungen auszusprechen. Dies wurde entsprechend als Konsequenz aus dem steigenden Bedarf an ICU-Kapazitäten (ICU/Intensive Care Unit) durch COVID-19 umgesetzt. Konkrete Strategien, wie diese Verschiebungen konkret kompensiert und Ausgleichskapazitäten geschaffen werden können, wurden zum damaligen Zeitpunkt nicht erörtert.

In jüngster Zeit wurden vermehrt Berichte über Defizite in der medizinischen Versorgung während dieser Zeit publiziert [2–7]. So wurden leere Warte- bzw. Behandlungszimmer bei niedergelassenen Ärzten genauso thematisiert wie abgesagte Verlaufsuntersuchungen bei chronischen Erkrankungen oder aufgeschobene Vorsorge- bzw. Früherkennungsmaßnahmen. Die langfristigen Auswirkungen dieser damals eingeschränkt angebotenen bzw. genutzten medizinischen Dienstleistungen lassen sich erst mit erheblicher Verzögerung beurteilen [7].

Die unmittelbaren Folgen für die stationäre Versorgung waren national wie auch global gravierend: Die Zahl der Krankenhausfälle in Deutschland ging zwischen Januar und September 2020 um 2,1 Mio. zurück (−15,5 %). In den Kalenderwochen zwei bis elf (6. Januar bis 15. März) lag die Patientenzahl noch auf dem Niveau der Vorjahre. Mit Beginn der zwölften Kalenderwoche (16. bis 22. März) nahm die Zahl der Patienten im Vergleich zum Vorjahreszeitraum um 24,6 % ab. Bis zur 15. Kalenderwoche (6. bis 12. April) sank die Fallzahl um 40,9 %, danach stieg sie wieder leicht an. Die Zahl der während dieser Zeit verschobenen operativen stationären und ambulanten Eingriffe in Deutschland lag vermutlich bei etwa 70.000 Eingriffen pro Woche [6]. Die COVIDSurg Collaborative berichtet, dass weltweit in den ersten 12 Wochen der Pandemie mindestens 30 Mio. elektive Operationen abgesagt wurden [5].

Die meisten Studien und Berichte zu dieser Thematik sind fächerübergreifend oder beziehen sich ganz allgemein auf die operative Leistungserbringung.

Um die Rahmenbedingungen der gefäßchirurgischen Versorgung in Deutschland während dieser Zeit zu erfassen, wurde vom Deutschen Institut für Gefäßmedizinische Gesundheitsforschung (DIGG) eine Umfrage initiiert, die sich an alle leitenden Gefäßchirurg*innen in Deutschland richtete. Zweck der Befragung war die Erfassung der gefäßchirurgischen Leistungserbringung in der Zeit von März 2020 bis Dezember 2020, sowie von logistischen und infrastrukturellen Veränderungen, die sich durch die pandemische Lage ergeben hatten. Hierbei lag der Fokus der Umfrage auf der möglichst realitätsnahen Abbildung der Versorgungssituation anhand der Einschätzung der leitenden Gefäßchirurg*innen.

## Studiendesign und Untersuchungsmethoden

In Zusammenarbeit mit der Deutschen Gesellschaft für Gefäßchirurgie und Gefäßmedizin (DGG) wurde das leitende ärztliche Personal sämtlicher gefäßchirurgischer Kliniken anhand einer Online-Recherche in Deutschland aufgefordert, an der Umfrage teilzunehmen. Diese wurde über die Online-Plattform surveymonkey® eingerichtet und war vom 10.09.2021 bis zu. 10.10.2021 zur Teilnahme freigeschaltet (siehe Fragebogen zur Umfrage). Die Beantwortung der Fragen erfolgte dabei anonym über den Einladungslink, nur vollständig erfasste Fragebögen wurden ausgewertet. Jeweils eine Befragung pro Einrichtung war möglich. Statistische Tests: Mann-Whitney-U-Test.

## Ergebnisse

93 vollständig beantwortete Umfrage-eCRF wurden während des Freischaltungszeitraums erfasst. Unvollständige Beantwortungen wurden nicht berücksichtigt. Der durchschnittliche Zeitaufwand für die Beantwortung der 28 Items wurde anhand der Bewertung der Umfrageplattform auf 6 min kalkuliert. Hinsichtlich der regionalen Verteilung der befragten Einrichtungen waren sämtliche Bundesländer bis auf Berlin, Bremen und Mecklenburg-Vorpommern vertreten. Die meisten Beantwortungen stammten aus Nordrhein-Westfalen (*n* = 20) und Baden-Württemberg (*n* = 15). 96,8 % der Teilnehmer (*n* = 90) waren in chefärztlicher oder leitender oberärztlicher Funktion tätig. 39 Einrichtungen, an denen die Teilnehmenden tätig waren, hatten die Versorgungsstufe I und II. 29 Kliniken hatten die Versorgungsstufe III. 15 Einrichtungen waren Universitätskliniken. 10 Einrichtungen waren Fachkrankenhäuser oder andere nicht näher klassifizierbare Einrichtungen. Lediglich in einem Fall wurde angegeben, dass an der Institution keine Behandlung von COVID-19-Patienten im Beobachtungszeitraum vorgenommen worden war. An 98,8 % der Einrichtungen war eine intensivmedizinische Versorgung von COVID-19-Patienten durchgeführt worden. Hierbei hatten 28 Einrichtungen eine spezielle COVID-19-ICU eingerichtet. In 4 Kliniken fand die Versorgung der COVID-Patienten ausschließlich auf der Operativen Intensivstation statt. Bei dem überwiegenden Teil der Einrichtungen fand dies jedoch auf einer interdisziplinären ICU statt (*n* = 63; 67,8 %). Bezogen auf das Krankheitsbild zeigte sich anhand der Aufnahmediagnosen, dass die überwiegende Anzahl der gefäßchirurgisch behandelten COVID-Patienten kardiovaskulär vorerkrankt war und an Diabetes mellitus sowie an Adipositas litt. Weitere patientenspezifische Parameter wurden nicht im Detail erhoben.

Hinsichtlich der veränderten Bettenkapazität wurde eine durchschnittliche pandemiebedingte Reduktion der Bettenkapazität um −30 % angegeben. Bezogen auf gefäßchirurgische Abteilungen schätzte die größte Gruppe der Befragten (38,7 %) die Reduktion der Bettenkapazität auf −25 bis −49 %. 6,5 % der Befragten gaben sogar eine Reduktion von bis zu −74 % an. 25,8 % gaben an, keinerlei Änderungen der Bettenkapazität festgestellt zu haben., in einem weiteren Viertel (24,7 %) der Kohorte waren es Bettenverluste von bis zu 25 %. Eine Minorität gab einen Zuwachs der Bettenkapazität an (4,3 %).

Hinsichtlich der Veränderung der personellen Besetzung zeigten sich die größten negativen Effekte beim Pflegepersonal. 54,8 % der Einrichtungen gaben an, dass vereinzelt originär der Gefäßchirurgie zugeordnete Pflegekräfte zur ausschließlichen Versorgung von COVID-19-Patienten abgeordnet wurden. 28,0 % der Teilnehmenden beschrieben die Abordnung der Pflegekräfte in der Größenordnung einer für die reguläre Patientenversorgung kritischen Anzahl. Eine Personalverschiebung bei ärztlichem gefäßchirurgischem Personal, die nach Einschätzung der Befragten einen für die Patientenversorgung kritischen Umfang hatte, wurde nur in 7,53 % der Fälle beschrieben, jedoch musste auch hier mehr als die Hälfte der Einrichtungen (54,8 %) ärztliches Personal zur ausschließlichen Versorgung von COVID-19-Patienten abordnen. Auf die Frage nach einer Veränderung des ärztlichen Personalschlüssels haben 80,7 % der Befragten keine Änderung der zur Verfügung stehenden Vollkraftstellen berichtet. 11,8 % haben eine in Zusammenhang mit der Pandemie stehende Kürzung um eine oder mehr Vollkraftstellen erfahren müssen.

**Figure 1:**
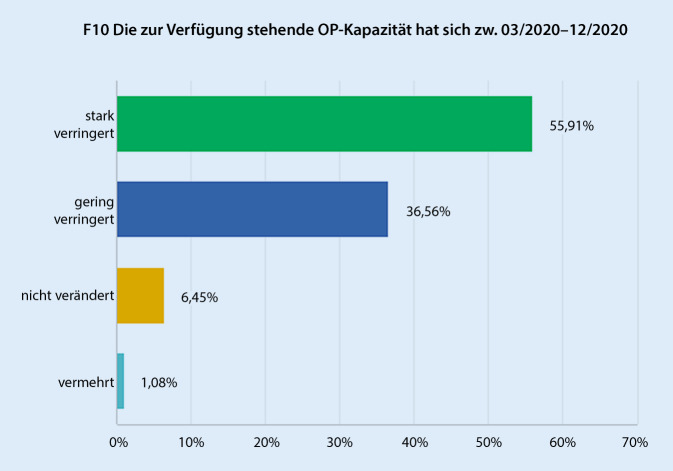

**Figure 2:**
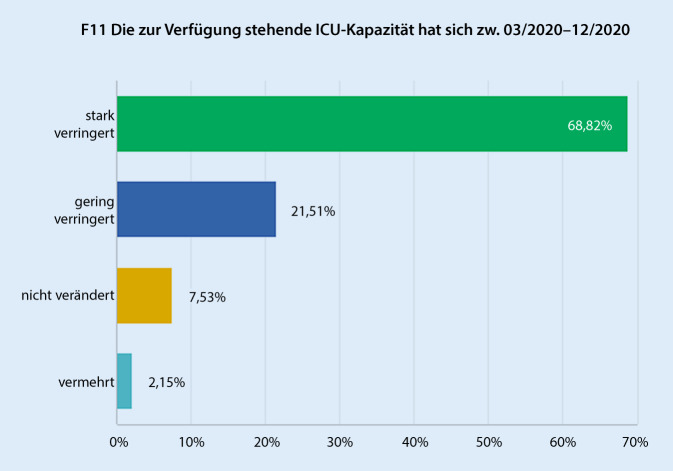

**Figure 3:**
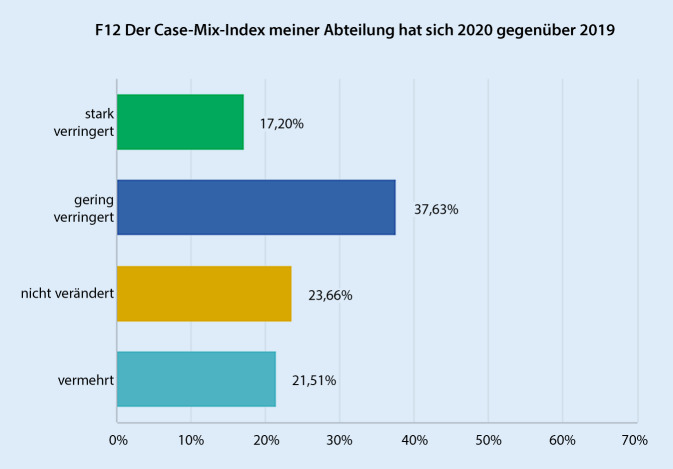

Die OP-Kapazität hat sich nach Einschätzung der Befragten in 55,9 % stark verringert (skaliert: „gering verringert“, „stark verringert“, „nicht verändert“, „vermehrt“), lediglich 7,5 % gaben keine negative Veränderung oder einen Zuwachs der OP-Kapazität an (Abb. 1). Korrespondierend hierzu gaben 68,8 % eine starke und 21,5 % eine geringe Reduktion der zur Verfügung stehenden ICU-Kapazität an (Abb. 2). Hinsichtlich des Case-Mix-Index (CMI) haben 54,8 % eine geringe bis starke Reduktion verzeichnet. Bei 23,7 % kam es zu keiner Veränderung. Eine Erhöhung des Case-Mix-Index beschrieben 21,5 % (Abb. 3). Für den entsprechenden aktuellen Zeitraum 2021 gaben 87,02 % an, dass diese personellen und strukturellen Veränderungen in gleichem oder geringerem Umfang weiterhin andauern. Mit „nein“ wurde die Frage nach dem Andauern dieser Veränderungen in 2021 von 12,9 % der Befragten beantwortet.

83 befragte Teilnehmende gaben retrospektiv betrachtet an, dass elektive gefäßchirurgische Operationen im Beobachtungszeitraum aufgrund der pandemischen Lage abgesagt wurden. 10 Kliniken hatten diesbezüglich keine Einschränkungen (10,8 %). Bei den betroffenen Einrichtungen waren die Unterschiede des Ausmaßes der Einschränkungen sehr deutlich. Insgesamt wurden nach den Angaben der Teilnehmer*innen 6805 elektive Eingriffe nicht durchgeführt oder um mehr als 3 Monate verschoben. Hier wurde nicht in Art und Umfang des Eingriffs unterschieden. Im Mittel waren dies 85 Eingriffe (Standardabweichung: 66,7) bei den betroffenen Kliniken.

**Figure 4:**
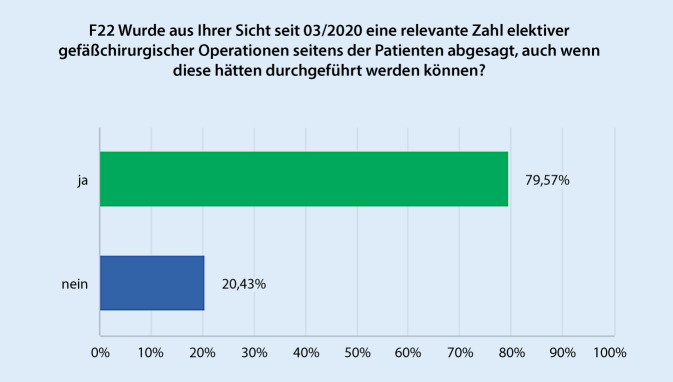

**Figure 5:**
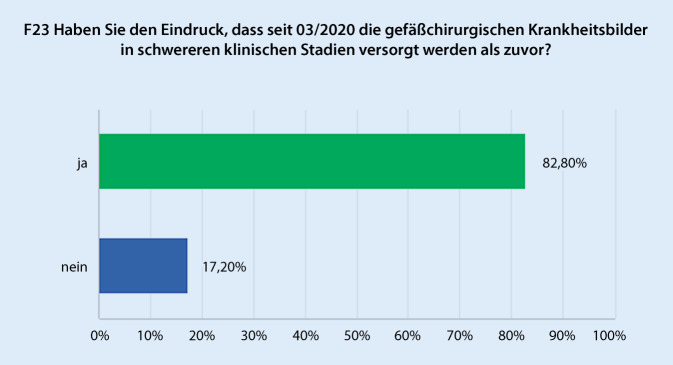

Hinsichtlich der subjektiven Einschätzung, inwieweit Eingriffe auf eigenen Wunsch des Patienten abgesagt wurden, obwohl diese hinsichtlich der Versorgungssituation im Beobachtungszeitraum hätten durchgeführt werden können, gaben 79,6 % der Befragten an, dass dies im jeweiligen Zentrum in relevantem Umfang geschehen ist (Abb. 4). Korrespondierend beschreibt eine deutliche Mehrheit (82,8 %), dass gefäßchirurgische Krankheitsbilder im Beobachtungszeitraum in relevant schwerwiegenderen klinischen Stadien behandelt wurden (Abb. 5). Dies spiegelt sich in einer Zunahme der Häufigkeit von Major- und Minoramputationen bei 62,4 % der Einrichtungen wider. Allerdings wurde ein häufigeres Auftreten von symptomatischen und rupturierten Aortenaneurysmen nur von 15,5 % der Befragten beobachtet.

Eine zeitgerechte Versorgung von Notfällen (6-h-Versorgungsfrist) hat sich nach Einschätzung von 44,1 % der Befragten relevant verschlechtert, 48,4 % sahen diesbezüglich keine Veränderung, 7,5 % konnten keine Angabe machen.

Ergänzend wurde erhoben, inwieweit sich nach Einschätzung der befragten Kolleg*innen die persönliche Belastung der ärztlichen Mitarbeitenden im Beobachtungszeitraum, bzw. mit Beginn der mit COVID-19 assoziieren Maßnahmen seit 03/2021 im Vergleich zum Vorjahreszeitraum entwickelt hat. Hier sehen 46,2 % eine relevante Zunahme der persönlichen Belastung, 19,4 % eine starke bis kritische Zunahme der persönlichen Belastung. 24,7 % konnte keine Veränderungen beobachten. 9,8 % geben an, dass sich der persönliche Belastungslevel der Mitarbeiter*innen reduziert hat. Hinsichtlich des Auftretens an Krankmeldungen, die nicht mit der COVID-19-Erkrankung an sich assoziiert waren, wurde von 35,48 % angegeben, dass im Beobachtungszeitraum eine relevante Zunahme (> 20 %) an Ausfalltagen im Vergleich mit dem Vorjahreszeitraum aufgetreten ist.

## Vergleich zwischen unterschiedlichen Versorgungsstufen

Um zu untersuchen, wie sich die erhobenen Parameter zwischen den Versorgungsstufen der befragen Einrichtungen unterschieden, wurden separat die Antworten der Gruppe „Versorgungsstufe III und Universitätsklinikum“, sowie „Versorgungsstufe I und II“ ausgewertet. Hier zeigte sich hinsichtlich der meisten Parameter kein signifikanter Unterschied. Auffallend war lediglich, dass Einrichtungen der Versorgungsstufen I und II verglichen mit Versorgungsstufe III und Universitätskliniken signifikant geringere Einbußen an ICU-Kapazität berichteten, die in der Einrichtung für operative Fächer zur Verfügung stehen („stark verringert“ 61,5 % vs. 75,0 %, *p* < 0,05), Tab. 1. Weiterhin konnte im Vergleich 2020 vs. 2019 bei den Einrichtungen einer geringeren Versorgungsstufe eine Zunahme des Case-Mix-Index (25,6 % vs. 18,2 %, *p* < 0,05) beobachtet werden, hinsichtlich der Veränderung der Case-Mix-Punkte gab es jedoch keine signifikanten Unterschiede zwischen diesen Gruppen.

**Table 1:** 

Parameter*		Versorgungsstufe I–II (%), *n* = 39	Versorgungsstufe III und Universitätskliniken (%), *n* = 44	*p*
OP-Kapazität	Stark verringert	56,4	59,1	> 0,05
	Gering verringert	33,3	34,1	> 0,05
	Nicht verändert	7,7	6,8	> 0,05
	Vermehrt	2,6	0	> 0,05
ICU-Kapazität	Stark verringert	**61,6**	**75,0**	**< 0,05**
	Gering verringert	23,1	20,5	> 0,05
	Nicht verändert	12,8	2,3	> 0,05
	Vermehrt	2,6	2,3	> 0,05

^a^ Nicht berücksichtigt wurden Fachkliniken und Einrichtungen, die keiner Versorgungsstufe gemäß eCRF zugeordnet wurden

## Diskussion

Die Versorgungssituation der meisten medizinischen Einrichtungen hat sich seit Beginn der COVID-19-Pandemie global verändert. Anlässlich der Jahrestagung der Deutschen Gesellschaft für Gefäßchirurgie und Gefäßmedizin 2021 wurde in Zusammenarbeit mit dem Deutschen Institut für Gefäßmedizinische Gesundheitsforschung gGmbH die vorliegende Umfrage durchgeführt und die Ergebnisse auf der Jahrestagung 2021 präsentiert. Als Beobachtungszeitraum wurde der Zeitraum zwischen März und Dezember 2020 gewählt, da insbesondere die Einschränkungen in der Anfangsphase der Erkrankung, sowie mittelfristigen Auswirkungen auf die Versorgungsrealität abgebildet wurden [1]. Entsprechend wurde bewusst ein längeres Intervall während der ersten Monate der Maßnahmen erfasst. Außerdem wurde, um eine bessere Vergleichbarkeit potenzieller Änderungen zum aktuellen Zeitpunkt zu ermöglichen, das Beobachtungsintervall von 03/2020 bis 12/2020 begrenzt. Der Fokus dieser Umfrage war, dass insbesondere Teilnehmende aus klinischen Zentren Ihre Einschätzung aufgrund der Wahrnehmungen der veränderten Versorgungssituation teilen und in diesem Sinne eine qualitative Bewertung der veränderten Umstände abgeben. Dies soll eine, neben den in der Literatur veröffentlichen Daten [5, 7], für die Gefäßchirurgie realitätsnahe Einschätzung der medizinischen und personellen Konsequenzen der Pandemie ermöglichen. Es konnten mit 93 vollständigen Beantwortungen 80 % der gefäßchirurgischen Zentren in Deutschland erfasst werden.

Auffallend war, dass 98,9 % dieser Einrichtungen COVID-19-Patienten behandelten. In der Diskussion der Ergebnisse an der Jahrestagung klärte sich, dass die einzige Einrichtung, die hier angab, keine COVID-Patienten zu versorgen, in einer Sondersituation eines Klinikträgers mit mehreren Standorten war, der die jeweiligen Standorte in COVID- und Nicht-COVID-Einrichtungen unterteilte. Dies stellt ein Beispiel für strukturelle Veränderungen als Anpassung auf seitens der Politik geforderte Bereitstellung von Versorgungskapazitäten dar.

Es zeigte sich, dass insbesondere verminderte Intensivkapazitäten und ein Mangel an Pflegepersonal, bzw. die Umwidmung von Pflegepersonal in explizite COVID-Behandlungsbereiche kritische Größen darstellten, die zu einer Reduktion der operativen Kapazitäten von Nicht-COVID-Bereichen führten. Vorangegangene Studien belegten, dass für COVID-19-Patienten, insbesondere auf Intensivstationen, ein wesentlich höherer personeller und apparativer Aufwand betrieben werden muss. Dies führt zu einem eklatant gesteigerten Verbrauch personeller und apparativer Ressourcen [8, 9], die wiederum unmittelbar eine Reduktion der operativen und ambulanten Versorgungskapazität bewirken. Ein vergleichsweise stärkerer Verlust von Intensivkapazitäten in Kliniken höherer Versorgungsstufe lässt sich durch eine Kumulation besonders kritisch kranker Patienten in Einrichtungen mit ARDS-Zentrum und ECMO-Betreuung erklären.

Die Mehrzahl der Befragten (54,8 %) berichteten im Vergleich zu 2019 über eine geringe bis starke Abnahme des Case-Mix-Index. Dies deckt sich mit Erhebungen, die für andere chirurgische Disziplinen gemacht wurden [10]. Man kann die Beobachtung korrelieren, dass 82,8 % berichten, dass Behandlungen in schweren klinischen Stadien erfolgten, bzw. somit eine Zunahme der Komplexität der klinischen Leistung vorlag. Hier sind eventuelle positive Effekte durch einrichtungsspezifische, in dieser Umfrage nicht deutlicher zu unterscheidende strukturelle Gegebenheiten möglich. Jedoch unterschieden nach „Versorgungsstufe der Einrichtung“ war eine Zunahme des Case-Mix-Index in Einrichtungen der Versorgungsstufe I und II deutliche. Man kann daraus schließen, dass hier die Beschränkung auf Notfalleingriffe und dringliche Operationen einen merklichen Effekt auf den Case-Mix-Index hatte, die Gesamtleistung also insgesamt wahrscheinlich nummerisch geringer, jedoch komplexer war, während in Einrichtungen mit höherer Versorgungsstufe und damit anzunehmender a priori höherer Komplexität der Behandlungen bzw. höherem Case-Mix-Index eine derartige Veränderung weniger ins Gewicht fällt.

Zusammenfassend lassen sich aufgrund dieser Umfrage keine reproduzierbaren Effekte auf die ökonomischen Auswirkungen der COVID-19-Pandemie und die Wirksamkeit des COVID-19-Krankenhausentlastungsgesetzes ableiten. Erste Untersuchungen legen jedoch nahe, dass die unselektive Einschränkung des elektiven Operationsbetriebs die Patientenversorgung und die Erlöskennzahlen signifikant beeinflusst und die einheitliche Freihaltepauschale zu einer unausgeglichenen Verteilung der finanziellen Hilfen abhängig von standortspezifischen Kriterien führt [10].

Aus dieser Erhebung kann abgeleitet werden, dass die Schwere der behandelten Krankheitsbilder zugenommen haben könnte. In der Literatur werden hierzu durchaus unterschiedliche Effekte aus den verschiedenen chirurgischen Disziplinen beschrieben. Manche Autoren sehen keinen signifikanten Unterschied in Morbidität und Mortalität chirurgischer Patienten während der Lockdown-Maßnahmen, während andere die hier beschriebenen Beobachtungen bestätigen [4, 10]. Deutlich wird jedoch, dass insbesondere die chirurgische Behandlung akuter Notfälle aufgrund zusätzlich implementierter Hygienemaßnahmen (Testung, Schleusung, Schutzkleidung, Masken etc.) signifikant verzögert wird („time to surgery“) [4]. Die hier berichtete Zunahme von Major- und Minoramputationen lässt sich als Ergebnisparameter für das Krankheitsbild der PAVK verstehen. Vor diesem Hintergrund wird vermutet, dass beispielsweise Patienten mit subakuten oder chronischen Durchblutungsstörungen, die primär als nicht unmittelbar vital bedroht angesehen werden, möglicherweise erst verspätet den Weg zum Spezialisten finden und infolgedessen ein ungünstigeres Behandlungsergebnis haben [11].

Die Einschätzung von über 80 % der Befragten, dass Eingriffe aufgrund von administrativen Vorgaben abgesagt wurden, obwohl diese aufgrund der Versorgungskapazität der Einrichtung hätten durchgeführt werden können, spricht dafür, dass die verfügten Maßnahmen in hohem Maße nicht an die epidemiologischen Rahmenbedingungen angepasst wurden.

Eine Untersuchung mit Fokus auf die Hernienchirurgie zeigte unter anderem, inwieweit seitens der Patienten Verständnis gegenüber der Absage oder Verschiebung einer Operation bestand. Insbesondere zu Beginn der pandemischen Einschränkungen bestand großes Verständnis der Patienten für Verzögerungen trotz subjektiver Dringlichkeit [2].

Dieser Effekt ist aus unserer Sicht insbesondere durch das frühe Stadium der Maßnahmen zu erklären und wird nicht von Dauer sein, da mit dem Fortdauern der Einschränkungen sowohl die medizinische Vertretbarkeit einer Aufschiebung als auch ein Verständnis seitens der Patient*innen abnehmen wird. Um insbesondere negativen Effekten einer Aggravation des Krankheitsstadiums und einer wachsenden persönlichen Frustration zu begegnen, schlagen Wilms et al. vor, die engmaschige klinische Kontrolle bei symptomatischen Patienten zu optimieren oder auch telemedizinische Konzepte zu etablieren, um innerhalb definierter Fenster einer operativen Kapazität prioritäten- und risikoorientiert Eingriffe planen zu können [2].

Nicht zu vernachlässigen ist außerdem die enorm gestiegene Belastung der ärztlichen und pflegerischen Mitarbeitenden durch die in Verbindung mit COVID-19 stehenden beruflichen Veränderungen. Dies bezieht sich sowohl auf den größeren administrativen hygienebedingten Mehraufwand als auch auf die persönlichen Lebensbedingungen während der der Pandemie. Hier spielen Faktoren wie Betreuung von Kindern und Angehörigen, Isolationsmaßnahmen im privaten Umfeld und potenzielle finanzielle Einbußen durch Kurzarbeit und Jobverlust eine Rolle [12]. Im Rahmen der VOICE-Studie wurden 2021 über 8000 Mitarbeitende im Gesundheitssektor befragt. Hier zeigte sich, dass mehr als 17 % des medizinischen Personals während der COVID-19-Pandemie 2020 unter psychischen Symptomen litt [13, 14]. Die Autoren identifizierten insbesondere das weibliche Geschlecht, einen Pflegeberuf und die direkte Betreuung von COVID-19-Patienten als Risikofaktoren. Diese Beobachtung konnte in der vorliegenden Umfrage bestätigt werden. Wir stellten sowohl einen deutlich gestiegenen persönlichen Belastungslevel der Mitarbeitenden als auch nicht unmittelbar durch COVID-19 bedingte Krankmeldungen in 35,38 % der Einrichtungen fest. Hier erscheint zwingend erforderlich, dass auf die Mehrbelastung der Mitarbeiter im Sinne einer nachhaltigen Personalpolitik reagiert wird und strukturierte Empfehlungen und Maßnahmen ergriffen werden, um Mitarbeiter angesichts der zunehmenden neuen Herausforderungen zu entlasten. Im Rahmen des Nationalen Netzwerks der Universitätsmedizin, bei dem auch die VOICE-Studie entstanden ist, ist eine Webseite entwickelt worden, die eine Datenbank mit Best-Practice-Interventionen anbietet [15]. Es geht um Projekte zur Förderung der psychischen Mitarbeitendengesundheit, wie Anreize zur Motivierung der Mitarbeitenden, kontextbezogene Veränderungen sowie konkrete Angebote psychosozialer Unterstützung [13]. Derartige Angebote und der Ausbau von Coping-Strategien sind sehr wichtig, um Mitarbeitende zu schützen. In erster Linie ist jedoch eine Verbesserung der tatsächlichen Arbeitsbedingungen, ein Ausbau personeller und apparativer Ressourcen und eine finanzielle Entlastung essenziell, wodurch einer vermehrten psychischen Belastung vorgebeugt werden kann.

## Limitationen dieser Studie

Da die Aussagen dieser auf der subjektiven Wahrnehmung der Befragten basieren, ist hier keine quantitative Beurteilung der erhobenen Parameter möglich. Weiterhin können aufgrund der erheblichen Varianz der lokalen und strukturellen Gegebenheiten der befragten Zentren Unschärfen hinsichtlich der einzelnen Parameter bestehen, die sich nicht weiter nachvollziehen lassen. Dies wurde zugunsten einer umfassenden Darstellung einer möglichst großen Schnittmenge gefäßchirurgischer Einrichtungen akzeptiert.

## Fazit für die Praxis

- Durch COVID-19 und korrespondierende Maßnahmen kam und kommt es zu relevanten Absagen und Verschiebungen von Operationen, einem Verlust an Kapazitäten und einer hohen Personalbelastung.
- Laut dieser Umfrage kam es zu einer verspäteten Versorgung und schwereren Stadien, obwohl in der Theorie Patient*innen hätten versorgt werden können.
- Betroffen sind alle Versorgungsstufen, größtenteils dauern diese Veränderungen an.
- Um diesen Missständen zu begegnen, sind strukturelle Abläufe, Patientenaufklärung und Priorisierung zu optimieren.
- Neue Konzepte wie z. B. Telemedizin und engmaschigere klinische Kontrolle sind ggf. sinnvoll.
- Infrastruktur für Notfallmanagement (COVID) darf im Alltag nicht die Versorgungsqualität der gefäßchirurgischen Patient*innen negativ beeinflussen!

## Supplementary Information




