# Supplementary material for: Care of vascular surgery patients during COVID-19: a Germany-wide survey
Source: Gefasschirurgie. 2022 Mar 4;27(4):274–81. [Article in German] doi: 10.1007/s00772-022-00871-8 (PMC8895360; doi:10.1007/s00772-022-00871-8)
Supplement: Supplementary file 1 [file 772_2022_871_MOESM1_ESM.pdf]

Versorgungsrealität gefäßchirurgischer Patienten während der COVID-19 Pandemie

Anonyme Umfrage

Informationen zum Datenschutz

Im Rahmen dieser Online-Befragung werden keine personenbezogenen Daten erhoben. Die Umfrage erfolgt absolut anonym, sodass kein Rückschluss auf einzelne Teilnehmende möglich ist. Gespeichert werden lediglich die Angaben, die Sie auf den folgenden Seiten dieser Befragung machen. Deren Verarbeitung unterliegt sowohl dem Bundesdatenschutzgesetz (BDSG) als auch der Datenschutz-Grundverordnung (DSGVO).

Die Ergebnisse der Studie werden anonymisiert und aggregiert ausgewertet. Im Rahmen der Aufarbeitung ist eine Publikation auf der Jahrestagung der DGG und im Journal GEFÄSSCHIRURGIE angedacht.

Die Teilnahme an der Umfrage ist freiwillig und kann jederzeit abgebrochen werden. Dazu müssen sie lediglich die Umfrage schließen und nicht speichern.

* 1. In welche Kategorie ist Ihr Klinikum eingeordnet?

- [ ] Versorgungsstufe I oder II
- [ ] Versorgungsstufe III
- [ ] Fachkrankenhaus
- [ ] Universitätsklinikum
- [ ] Keine der oben genannten

* 2. Ihre Klinik liegt in welchem Bundesland?

- [ ] Baden-Württemberg
- [ ] Bayern
- [ ] Berlin
- [ ] Brandenburg
- [ ] Bremen
- [ ] Hamburg
- [ ] Hessen
- [ ] Mecklenburg-Vorpommern
- [ ] Niedersachsen
- [ ] Nordrheinwestfalen
- [ ] Rheinland-Pfalz
- [ ] Saarland
- [ ] Sachsen
- [ ] Sachsen-Anhalt
- [ ] Schleswig-Holstein
- [ ] Thüringen

* 3. Ihre Funktion in Ihrer Klinik

- [ ] Facharzt / Oberarzt
- [ ] Oberarzt mit Leitungsfunktion
- [ ] Chefarzt / Direktor
- [ ] Keine der oben genannten

* 4. Unser Klinikum behandelt Pat mit COVID 19 Infektionen

- [ ] Ja
- [ ] Nein

* 5. Es findet auch eine intensivmedizinische Behandlung von COVID Pat. statt.

- [ ] Nein
- [ ] nur in der chirurgischen ICU
- [ ] nur in der medizinischen ICU
- [ ] Sonstige ICU
- [ ] in einer interdisziplinären ICU
- [ ] in einer speziellen COVID ICU

* 6. Veränderung der Bettenkapazität ihres Gesamtklinikums zwischen 3/2020 und 12/2020 (Abzgl. vorher geplanter Veränderungen)

- 30% keine Änderung + 30%

* 7. Veränderung der Bettenkapazität Ihrer gefäßchirurgischen Abteilung zwischen 03/2020 und 12/2020

- [ ] - 100%
- [ ] - 75-99%
- [ ] -50-74%
- [ ] -25-49%
- [ ] -1-24%
- [ ] keine Änderung
- [ ] +1-25%
- [ ] >25%

* 8. Transfer von Pflege-Personal Ihrer Abteilung ausschließlich zur Behandlung von COVID-19 (zw. 03/2020 - 12/2020)

- [ ] ja; vereinzelt
- [ ] ja; kritische Menge
- [ ] nein

* 9. Transfer von ärztlichem Personal Ihrer Abteilung ausschließlich zur Behandlung von COVID-19 (zw. 03/2020 - 12/2020)

- [ ] ja; vereinzelt
- [ ] ja; kritische Menge
- [ ] nein

* 10. Die zur Verfügung stehende OP Kapazität hat sich zw. 03/2020-12/2020

- [ ] stark verringert
- [ ] gering verringert
- [ ] nicht verändert
- [ ] vermehrt

* 11. Die zur Verfügung stehende ICU Kapazität hat sich zw. 03/2020-12/2020

- [ ] stark verringert
- [ ] gering verringert
- [ ] nicht verändert
- [ ] vermehrt

* 12. Der casemix Index meiner Abteilung hat sich 2020 gegenüber 2019

- [ ] stark verringert
- [ ] gering verringert
- [ ] nicht verändert
- [ ] erhöht

* 13. Die casemix Punkte meiner Abteilung haben sich 2020 gegenüber 2019

- [ ] stark verringert
- [ ] gering verringert
- [ ] nicht verändert
- [ ] erhöht

* 14. Dauern die Veränderungen für Ihre Abteilung in 2021 an.

- [ ] ja, in geringerem Umfang
- [ ] ja, in gleichem Umfang
- [ ] Nein
- [ ] Keine der oben genannten

* 15. Wurde der ärztliche Personalschlüssel zur gefäßchirurgischen Versorgung für das Jahr 2021 verändert?

- ☐ Personalschlüssel (VK-Stellen) zur gefäßchirurgischen Versorgung wurde seitens des Arbeitgebers um 1 VK reduziert oder nicht nachbesetzt
- ☐ Personalschlüssel wurde um 2 VK reduziert oder nicht nachbesetzt
- ☐ Personalschlüssel wurde um 3 oder mehr VK reduziert oder nicht nachbesetzt
- ☐ keine Änderung
- ☐ Personalschlüssel (VK-Stellen) wurde erhöht
- ☐ Keine der oben genannten

16. Wieviele gefäßchirurgische Operationen an COVID-19 positiv getesteten Patient*innen wurden 2020 in Ihrer Klinik insgesamt durchgeführt?

[ ]

17. Bitte schätzen Sie die Anzahl der mit COVID-19 assoziierten und gefäßchirurgisch versorgten thrombotischen Gefäßverschlüsse

[ ]

18. Welcher Anteil (%) der gefäßchirurgisch operierten und an COVID-19 erkrankten Patient*innen hatte einen BMI > 30 Kg/m2

0% 50% 100% [ ]

19. Welcher Anteil (%) der gefäßchirurgisch operierten an COVID-19 erkrankten Patient*innen war kardiovaskulär vorerkrankt?

0% 50% 100% [ ]

20. Welcher Anteil (%) der gefäßchirurgisch operierten an COVID-19 erkrankten Patient*innen litt unter Diabetes mellitus Typ I oder II?

0% 50% 100% [ ]

* 21. Wieviele elektive gefäßchirurgische Operationen wurden in Ihrer Klinik seit 03/2020 aus Kapazitätsgründen abgesagt oder um mehr als 3 Monate verschoben?

[ ]

* 22. Wurde aus Ihrer Sicht seit 03/2020 eine relevante Zahl elektiver gefäßchirurgischer Operationen seitens der Patienten abgesagt, auch wenn diese hätten durchgeführt werden können?

- [ ] ja
- [ ] nein

* 23. Haben Sie den Eindruck, dass seit 03/2020 die gefäßchirurgischen Krankheitsbilder in schwereren klinischen Stadien versorgt werden als zuvor?

- [ ] ja
- [ ] nein

* 24. Hat seit 03/2020 die Häufigkeit von Major- und Minoramputationen zugenommen?

- [ ] ja
- [ ] nein

* 25. Hat die Häufigkeit symptomatischer oder rupturierter Aortenaneurysmen seit 03/2020 zugenommen?

- [ ] ja
- [ ] nein

* 26. Hat sich aus Ihrer Sicht seit 03/2020 die zeitgerechte Versorgung von Notfällen verschlechtert?

- [ ] ja
- [ ] nein
- [ ] mir nicht bekannt

* 27. Wie schätzen Sie die persönliche Belastung ihrer Mitarbeiter*innen seit 03/2020 ein?

- ○ Hat sich reduziert
- ○ Hat sich nicht verändert
- ○ Hat zugenommen
- ○ Hat stark zugenommen

* 28. Gab es seit 03/2020 bei Ihren Mitarbeiter*innen vermehrt Krankmeldungen, die nicht unmittelbar mit COVID-19 assoziiert waren?

- [ ] ja
- [ ] nein
- [ ] unbekannt
